# Tophaceous gout in the lumbar spinal canal mimicking epidural spinal tumor in a male adolescent: A case report

**DOI:** 10.1097/MD.0000000000043661

**Published:** 2025-08-15

**Authors:** Juan Chen, Min Li, Fanyu Zhao, Xin Xiang

**Affiliations:** a The Department of Rheumatology, Minzu Hospital of Guangxi Zhuang Autonomous Region, Nanning, China; b The Department of Radiology, The Second Affiliated Hospital, Guangxi Medical University, Nanning, China; c The Department of Radiology, Minzu Hospital of Guangxi Zhuang Autonomous Region, Nanning, China.

**Keywords:** adolescent, case report, gout tophus, lumber spine, spinal gout

## Abstract

**Rationale:**

Spinal gout is a rare occurrence, with varied symptoms based on the location of urate deposits, and diagnosis is typically delayed. Herein, we present a unique case of juvenile lumbar spinal gout.

**Patient concerns:**

A 16-year-old male patient was admitted to our hospital because of lower back pain for 11 days. histopathological evaluation identified gouty tophus in the lumbar spinal.

**Diagnoses:**

This patient was diagnosed with Tophaceous gout in the lumbar spinal.

**Interventions:**

Following laboratory and imaging assessment, the patient underwent surgery, and histopathological evaluation identified gouty tophus. Post surgery, the patient experienced a gradual improvement in symptoms, and he recovered well without any complications.

**Outcomes:**

During follow-up, the patient’s serum uric acid level was 354 µmol/L. Lumbar magnetic resonance imaging examination showed postoperative changes, with L5 anterior spondylolisthesis and L4/5, L5/S1 disc bulging.

**Lessons:**

Lumbar spinal gout is quite rare, and may go unnoticed by physicians. It is accompanied with varying manifestations. If lumbar spinal gout patients present with neurological symptoms or an indeterminate lesion, then surgical intervention can markedly improve symptoms and/or clarify diagnosis.

## 1. Introduction

Gout is a highly prevalent form of inflammatory arthritis, which induces pain and swelling in joints, and can progress to form persistent destructive joint disease.^[1]^ More recently, gout incidences have risen drastically among adolescents, partly due to alterations in diet, however, there are limited reports on the influence of gout on the axial skeleton. Moreover, the documented prevalence of lumber spinal gout tophus among adolescents is relatively low. Herein, we present a case of lumber spinal gout tophus in a male adolescent with preserved renal function to increase awareness of this condition.

## 2. Case presentation

A 16-year-old male sought treatment at our hospital for persistent right side lower back pain, which exacerbated during activities. He was immediately hospitalized for treatment, and was released upon recovery after 11 days. The patient also reported pain in the right popliteal fossa, alongside no history of fever, numbness, spinal injury, weight loss, or night sweats. His gout history spanned >2 months, and, at admission, he complained of first metatarsophalangeal joint pain and swelling, which was irregularly treated. The patient had no positive familial gout history.

Upon physical examination, we identified marked redness, swelling, and adiposity of the skin around the first metatarsophalangeal joint (body mass index = 31.8 kg/m^2^). The patient exhibited no other positive neurological symptoms. According to laboratory examination, the circulating uric acid content was 525 µmol/L, erythrocyte sedimentation rate was 36 mm, and C-reactive protein level was 4.21 mg/L. A complete blood count upon admission showed 11.21 × 10^9^/L white blood cells and 0.86 neutrophil percentage. The liver function and urinalysis appeared normal. The main laboratory results are shown in Table 1.

**Table 1:** Laboratory characteristics at admission.

Component	Value	Reference range	Interpretation
Blood chemistry			
Serum creatinine (µmol/L)	67	50–77	Normal
Blood urea nitrogen (mmol/L)	3.87	2.9–8.2	Normal
Uric acid (µmol/L)	525	208–428	High
K (mmol/L)	4.32	3.5–5.3	Normal
Na (mmol/L)	138.8	137–147	Normal
Cl (mmol/L)	100.6	96–108	Normal
Ca (mmol/L)	2.26	2.1–2.55	Normal
Serum albumin (g/L)	40.2	32–45	Normal
C-reactive protein (mg/L)	4.21	0–1	High
Erythrocyte sedimentation rate (mm)	36	0–15	High
Hematology			
White blood cells (×10^9^/L)	11.21	3.5–9.5	High
Neutrophils percentage (%)	0.86	0.4–0.75	High
Hemoglobin (g/L)	117	130–175	Low
Platelets (×10^9^/L)	448	125–350	High
Urinalysis			
Urinary protein	Negative	Negative	Normal
Red blood Cells (n/HP)	0	0–2	Normal
White blood Cells (n/HP)	0–3	0–3	Normal
Sugar sediment	Negative	Negative	Normal

**Figure 1.**
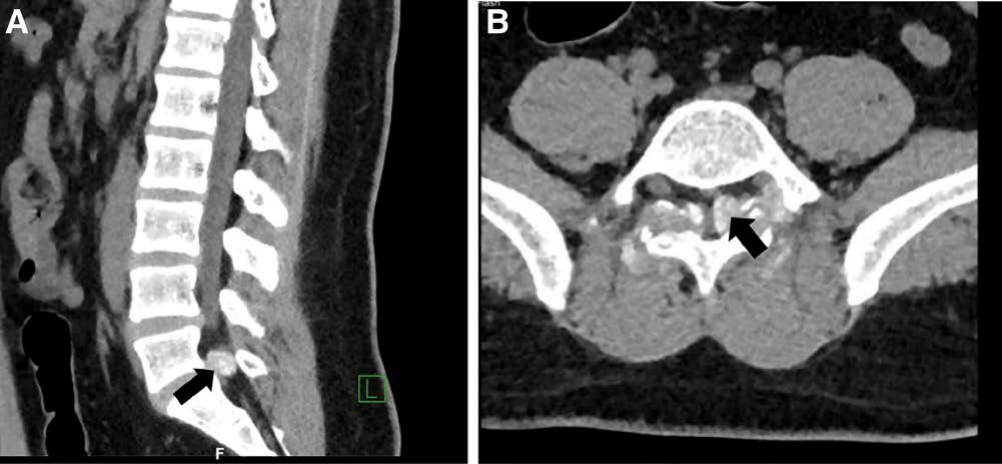
Computed tomography images of the lumbar spine. Note: images revealing partial lesions, round mass within the spinal canal at L5/S1 (indicated by the arrow in the sagittal tomogram [A]); insect erosion and destruction of the bilateral patellar bone in the adnexa of L5, as well as the bilateral involvement of the lower articular processes at L4 and the bilateral articular processes at S1, Corresponding segmental spinal stenosis (marked by an arrow in [B]).

Computed tomography revealed bilateral pedicle discontinuity at L5. Additionally, we observed insect erosion and destruction of the bilateral patellar bone in the adnexa of L5, along with the bilateral involvement of the lower articular processes at L4 and the bilateral articular processes at S1. We also identified soft tissue masses, and the lesions extended to the vertical spinal muscles on both sides, thereby causing secondary segmental spinal stenosis (Fig. 1).

**Figure 2.**
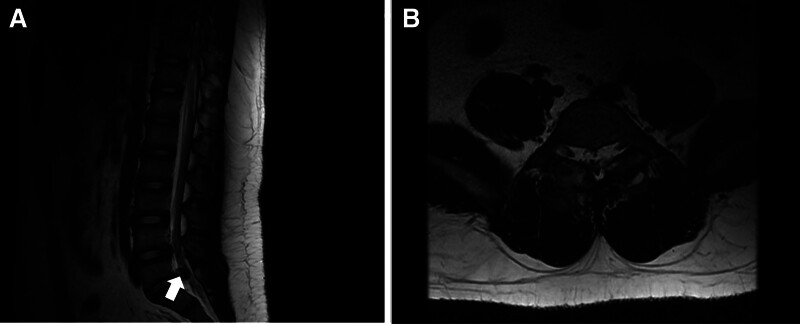
A lumbar spine MRI depicting abnormal signals at L5, slightly reduced signals on T1WI and T2WI, and slightly elevated lipid pressure sequences on T2 (both signals were unclear boundaries, and corresponded to spinal cord compression, indicated by an arrow). MRI = magnetic resonance imaging.

A lumbar spine magnetic resonance imaging (MRI) examination revealed abnormal signals on both sides of L5, slightly reduced signals on T1WI and T2WI, and slightly elevated lipid pressure sequences on T2; both signals were unclear boundaries, ranging from 3.4 cm × 3.0 cm × 3.8 cm to 2.8 cm × 1.8 cm × 4.7 cm, with discontinuous bilateral pedicle cortical bone in L5. Lesions were present in the bilateral lower articular process of the L4 cone, the upper bilateral articular process of the S1 vertebral body and the right spinous muscle, and the corresponding spinal cord appeared compressed. The L4/5 and L5/S1 intervertebral discs bulged peripherally, and the dural sac was compressed at the corresponding level, with the narrowing of the spinal canal at the corresponding level (Fig. 2).

Upon admission of patient, we conducted surgical intervention whereby we noticed significant destruction of the spinous process of L5, lamina and facet joints, as well as portion of the L4 lamina, alongside thickened ligamenta flavum and surrounding lesions. In particular, white powder was evident within the paraspinal muscles of L5/S1. In addition, bone destruction and white powdery lesions were present within the L5 pedicles as well as the upper and lower articular processes. The ligamentum flavum between the L4 and S1 was substantially thickened, and white powdery lesions were spread throughout the ligamentum. There was a 4 × 2 × 1 cm epidural mass within the spinal canal, the corresponding segment of the spinal canal appeared narrow, and the nerve root was obviously compressed. Consequently, we conducted nerve root canal decompression, and the corresponding segments of adherent nerve roots were released.

**Figure 3.**
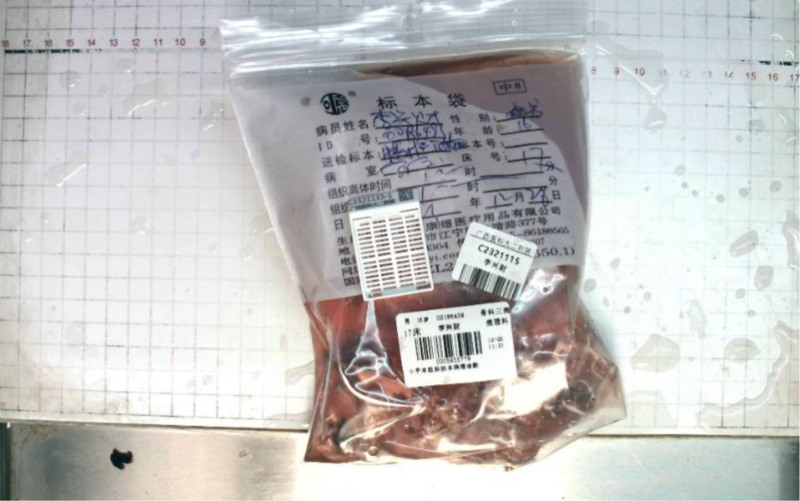
An image of the lumbar lesion depicting gray-red tissue totaling 9.3 cm × 7.1 cm × 2.5 cm in size.

**Figure 4.**
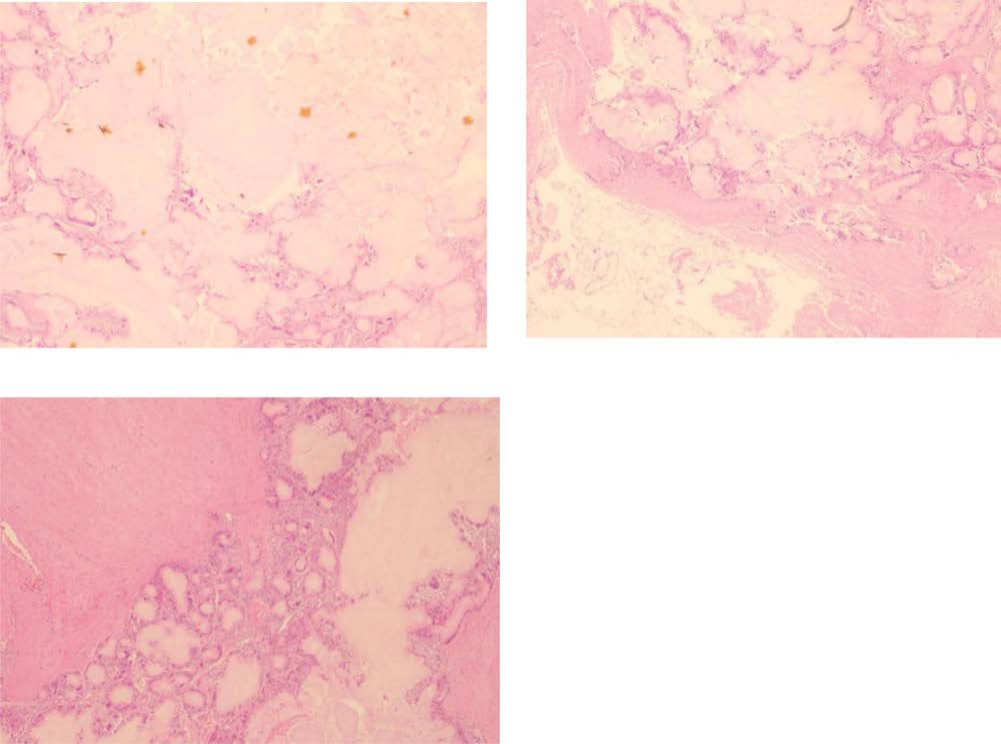
Microscopic specimen evaluation depicting gouty tophi, featuring an amorphous proteinaceous core and radial crystals, surrounded by fibroblasts, lymphocytes, and multinucleated giant cells, which was consistent with a gouty tophus diagnosis.

The excised tissue (Fig. 3) underwent fixation in 10% formalin for pathological evaluation. Microscopic assessment identified gouty tophi, featuring an amorphous proteinaceous core and radial crystals, surrounded by fibroblasts, lymphocytes, and multinucleated giant cells, which was consistent with a gouty tophus diagnosis (Fig. 4).

**Figure 5.**
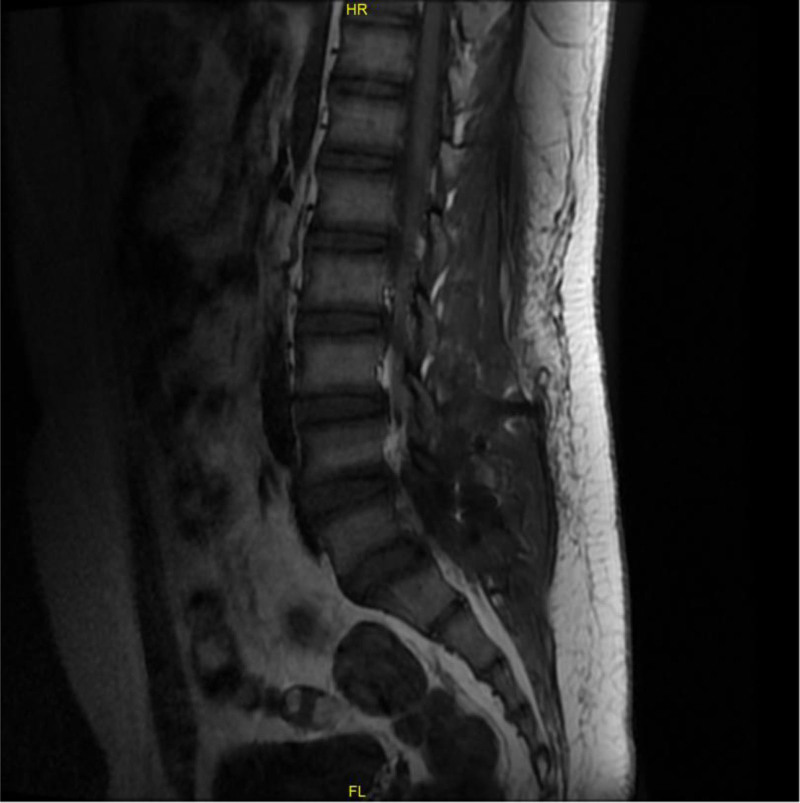
During follow-up, lumbar MRI examination showed postoperative changes, with L5 anterior spondylolisthesis and L4/5, L5/S1 disc bulging. MRI = magnetic resonance imaging.

Following surgical intervention, the patient experienced improved symptoms, and recovered completely without any complications. Post surgery, the patient experienced a gradual improvement in symptoms, and he recovered well without any complications. During follow-up, the patient’s serum uric acid level was 354 µmol/L. Lumbar MRI examination showed postoperative changes, with L5 anterior spondylolisthesis and L4/5, L5/S1 disc bulging (Fig. 5).

## 3. Discussion

Gout is a highly prevalent metabolic disorder brought on by urate accumulation. However, its involvement of the lumbar spine is quite uncommon, particularly among adolescents. Herein, we present the case of a 16-year-old male patient, who received gouty tophi diagnosis in the lumbar spine via biopsy. The patient reported lumbar pain as the only symptom. Earlier studies suggested^[2]^ that majority of the lumbar spine-based gouty tophus occurs among individuals between 60 and 69 years of age. The clinical manifestation can vary depending on the location of urate deposition, and can range between back pain, limb radiation pain and limb weakness, or certain neurological dysfunction, such as paresthesia and radiculopathy. In case of our patient, there was low back pain, with nonspecific clinical manifestations, which required delineation from other diseases, such as, spinal arthritis, and so on. Our patient reported a history of gout, and was suffering from pain and swelling of the metatarsal joint of the first foot in the right foot, which provided a strong clue. Earlier reports suggested that 24.6% of lumbar spinal gout patients do not have a history of gout or hyperuricemia.^[3]^ Therefore, when a patient complains of chronic lower back pain, gout must be considered, particularly, if the patient exhibits normal circulating uric acid level and no gout history.

Hyperuricemia and gout in adolescents typically indicate autosomal dominant tubular interstitial nephropathy (ADTKD).^[4]^ Among these patients, the fraction of uric acid released by uromodulin-associated ADTKD is generally reduced (<5%), which promotes early hyperuricemia and gout, and there is also a family history of kidney disease. Our patient exhibited normal renal function and adequate uric acid excretion. Moreover, he denied a family history of kidney disease, thus, other conditions were considered.

Hypoxanthine-guanine phosphoribosyl transferase (HPRT) deficiency causes hyperuricemia and gout, along with severe neurological disorders, namely, dance athetosis, mental retardation, and self-injurious behavior.^[5]^ In several investigations,^[6]^ young individuals only exhibit gout manifestations, with mild or absent neurological dysfunction. This partial HPRT deficiency suggests that clinical symptoms, HPRT activity, adenine phosphoribosyl transferase activity, and purine catabolism together facilitate diagnosis. Unfortunately, for this report, we did not have access to the equipment needed to perform these tests.

Prior studies reported that gout in adolescents is strongly linked to obesity, genetic background, and uric acid overproduction.^[7]^ Obesity stimulates the overproduction and insufficient excretion of uric acid, which results in hyperuricemia and gout. Multiple genome-wide analyses demonstrated association of certain uric acid-regulating genes, namely, SLC22A12, SLC2A9, ABCG2, SLC22A11, SLC22A13, and SLC17A1 with this disease.^[8]^ ABCG2 is ubiquitously expressed in renal tubules and intestines. Its dysregulation causes insufficient uric acid excretion, which accelerates hyperuricemia and gout formation. Moreover, severe ABCG2 dysfunction particularly increases early gout incidence.^[9]^ A genome-wide sequencing of an adolescent gout cohort identified rs12887440 (RCOR1) and rs35213808 (FSTL5-MIR4454) as risk loci for early-onset gout. RCOR1 is a major regulator of inflammation and immune responses. REST Corepressor 1 (RCOR1 or CoREST) is a protein that interacts with the C-terminal domain of suppressor element − 1 silenced transcription factor (REST). Additionally, it is intricately linked to gout formation among the G-H group (gout vs individuals with hyperuricemia), and is known to regulate numerous immune and inflammatory responses. Rcor1 depletion impairs T-bet, IL-2, and IFN-γ promoter recruitment by FOXP3-dependent CoREST complexes, which damages Treg function in vivo and elicits immune responses. These evidences suggest that the RCOR1 site possesses pro-inflammatory activity, and facilitates the progression from hyperuricemia to gout. During adolescence, gout- and immune-associated gene variants may increase sensitivity to urate crystal-driven inflammatory responses, thereby accelerating gout onset.^[10]^

Furthermore, several investigations identified a 869 T/C polymorphism of the transforming growth factor 1 gene, which is linked to gouty tophus deposition.^[11]^ The underlying mechanism involves the following: the aforementioned polymorphism induces TGF- 1 dysfunction. However, the inflammatory response is not fully terminated. Therefore, endothelial cells become activated, and some neutrophils infiltrate, which results in the activation of white blood cells and persistence of chronic inflammation, which eventually leads to gouty tophus formation.

The critical association between differential gene expression and lumbar spine gouty tophus formation required further exploration. Our patient had no familial gout history, yet he experienced gout attacks and developed lumbar gout stones during adolescence. This is highly indicative of underlying genetic variants regulating gout etiology.

Notably, the underlying network of gouty tophi etiology is poorly elucidated. Based on some scholars, gout may be the result of poor vascularization, which generates the optimal conditions for gout formation.

Hyperlipidemia, obesity, and minimal physical activity can generate significant obstruction in the small-caliber vascular system in the lumbar spine. This, in turn, can drastically reduce filtration of uric acid deposited in the area.^[12]^ Among the factors enabling uric acid deposition and tophi formation are acidic pH, matrix composition of the articular cartilage and low oxygen microenvironment. Our patient was obese, and conducted minimal physical activity, which, unfortunately, provided the optimal microenvironment for uric acid deposition. Therefore, we suspect that a metabolic disorder can significantly contribute to uric acid deposition.

At present, there are no standard diagnostic criteria for lumbar spinal tophaceous gout formation. Moreover, the imaging manifestations of lumbar gout on plain computed tomography scan are nonspecific, and the typically associated abnormalities include lumbar spondylolisthesis, degenerative alterations, and so on. Louie et al^[13]^ suggested that gout diagnosis may be made via MRI, depending on the T1 and T2-weighted image signal intensity. In their report, gout exhibited a reduced signal on MRI T1-weighted images and enhanced signal on MRI T2-weighted images. Unfortunately, with this information alone, there is no way to delineate gouty tophi from other diseases, namely, infectious lesions, epidural abscesses, rheumatoid arthritis, and metastatic diseases. Therefore, MRI was suggested. Our patient’s lumbar MRI indicated neoplasticity. Scholars speculate^[14]^ that, owing to the vascularized nature of gouty tophi, it often appears as low-intensity, uniform masses affecting in MRI T1 and T2-weighted images. Therefore, an imaging diagnosis of lumbar gout is rather challenging.

## 4. Conclusion

Lumbar spinal gout is a unique condition that may go unnoticed due to its variable and, sometimes, nonspecific clinical manifestation. In case of patients with lumbar spinal gout and neurological symptoms or an indeterminate lesion, surgical intervention can strongly benefit patient outcome and confirm disease diagnosis.

## Acknowledgments

The authors would like to thank the staff of The Department of Rheumatology, Minzu Hospital of Guangxi Zhuang Autonomous Region (Nanning, China) and The Department of Radiology, the Second Affiliated Hospital, Guangxi Medical University (Nanning, China).

## Author contributions

**Conceptualization:** Juan Chen, Min Li.

**Data curation:** Juan Chen, Min Li.

**Investigation:** Fanyu Zhao.

**Methodology:** Juan Chen, Min Li.

**Supervision:** Xin Xiang.

**Writing – original draft:** Juan Chen.

**Writing – review & editing:** Xin Xiang.
